# Circular RNA ZNF800 (hsa_circ_0082096) regulates cancer stem cell properties and tumor growth in colorectal cancer

**DOI:** 10.1186/s12885-023-11571-1

**Published:** 2023-11-10

**Authors:** Vimalan Rengganaten, Chiu-Jung Huang, Mong-Lien Wang, Yueh Chien, Ping-Hsing Tsai, Yuan-Tzu Lan, Hooi Tin Ong, Shih-Hwa Chiou, Kong Bung Choo

**Affiliations:** 1 Centre for Stem Cell Research, Universiti Tunku Abdul Rahman, 43000 Kajang, Selangor Malaysia; 2 Postgraduate Program, M. Kandiah Faculty of Medicine and Health Sciences, Universiti Tunku Abdul Rahman, 43000 Kajang, Malaysia; 3 Institute of Pharmacology, National Yang Ming Chiao Tung University, Taipei, 11221 Taiwan; 4 Department of Animal Science & Graduate Institute of Biotechnology, Chinese Culture University, Taipei, 11221 Taiwan; 5 School of Medicine, National Yang Ming Chiao Tung University, Taipei, 11221 Taiwan; 6 Department of Medical Research, Taipei Veterans General Hospital, Taipei, 11221 Taiwan; 7 Institute of Food Safety and Health Risk Assessment, National Yang Ming Chiao Tung University, Taipei, 11221 Taiwan; 8 Division of Colon & Rectal Surgery, Department of Surgery, Taipei Veterans General Hospital, Taipei, 11221 Taiwan; 9 Centre for Cancer Research, Universiti Tunku Abdul Rahman, 43000 Kajang, Selangor Malaysia; 10 Department of Preclinical Sciences, M. Kandiah Faculty of Medicine and Health Sciences, Universiti Tunku Abdul Rahman, Sg Long, 43000 Kajang, Selangor Malaysia

**Keywords:** circZNF800-miRNA-mRNA regulation, Cancer stem cells, Colorectal cancer, Tumorigenesis, CRISPR Cas13d knockdown

## Abstract

**Background:**

Cancer stem cells form a rare cell population in tumors that contributes to metastasis, recurrence and chemoresistance in cancer patients. Circular RNAs (circRNAs) are post-transcriptional regulators of gene expression that sponge targeted microRNA (miRNAs) to affect a multitude of downstream cellular processes. We previously showed in an expression profiling study that circZNF800 (hsa_circ_0082096) was up-regulated in cancer stem cell-enriched spheroids derived from colorectal cancer (CRC) cell lines.

**Methods:**

Spheroids were generated in suspension spheroidal culture. The *ZNF800* mRNA, pluripotency stem cell markers and circZNF800 levels were determined by quantitative RT-PCR. CircZNF800-miRNA interactions were shown in RNA pulldown assays and the miRNA levels determined by stem-loop qRT-PCR. The effects of circZNF800 on cell proliferation were tested by EdU staining followed by flowcytometry. Expression of stem cell markers CD44/CD133, Lgr5 and SOX9 was demonstrated in immunofluorescence microscopy. To manipulate the cellular levels of circZNF800, circZNF800 over-expression was achieved via transfection of in vitro synthesized and circularized circZNF800, and knockdown attained using a CRISPR-Cas13d-circZNF800 vector system. Xenografted nude mice were used to demonstrate effects of circZNF800 over-expression and knockdown on tumor growth in vivo*.*

**Results:**

CircZNF800 was shown to be over-expressed in late-stage tumor tissues of CRC patients. Data showed that circZNF800 impeded expression of miR-140-3p, miR-382-5p and miR-579-3p while promoted the mRNA levels of *ALK/ACVR1C, FZD3* and *WNT5A* targeted by the miRNAs, as supported by alignments of seed sequences between the circZNF800-miRNA, and miRNA-mRNA paired interactions. Analysis in CRC cells and biopsied tissues showed that circZNF800 positively regulated the expression of intestinal stem cell, pluripotency and cancer stem cell markers, and promoted CRC cell proliferation, spheroid and colony formation in vitro*,* all of which are cancer stem cell properties. In xenografted mice, circZNF800 over-expression promoted tumor growth, while circZNF800 knockdown via administration of CRISPR Cas13d-circZNF800 viral particles at the CRC tumor sites impeded tumor growth.

**Conclusions:**

CircZNF800 is an oncogenic factor that regulate cancer stem cell properties to lead colorectal tumorigenesis, and may be used as a predictive marker for tumor progression and the CRISPR Cas13d-circZNF800 knockdown strategy for therapeutic intervention of colorectal cancer.

**Supplementary Information:**

The online version contains supplementary material available at 10.1186/s12885-023-11571-1.

## Background

Cancer stem cells constitute a rare population in the heterogeneous cell population in tumors [1, 2]. It has been proposed that cancer stem cells are derived from normal stem cells residing in tissues or organs on assaults by mutations, or stem cell-like properties are acquired in terminally differentiated cells induced by de-differentiation mutations [2, 3]. Cancer stem cells are characterized by enhanced self-renewal abilities and tumorigenic properties, including unhindered cell division capacities [2]. Clinically, cancer stem cells contribute to metastasis, increased tumor aggressiveness, recurrence and chemo- and irradiation-resistance in therapeutic treatment of cancer patients [3, 4].

In colorectal cancer (CRC), the study model of this work, oncogenic mutations in the intestinal stem cell (ISC) population give rise to CRC stem cells to participate in CRC tumorigenesis [2, 5]. Introducing adenomatous polyposis coli mutations reportedly lead to adenoma only in LGR5^+^ (leucine-rich repeat containing G protein-coupled receptor 5) stem cells, which resembles ISC, but not in LGR5^−^ cell population, marking LGR5 a cancer stem cell marker in CRC [6]. The unhindered CRC stem cell proliferation as a result of gain-of-function mutations in the intestinal stem cells in the crypts of intestines has become a major challenge for therapeutic interventions [7, 8].

The rarity of cancer stem cells is often addressed using cancer cell-derived spheroids, which are enriched in cancer stem cells and are a convenient system for studying cancer stem cell properties and for elucidating the role of cancer stem cells in the tumorigenesis process [9]. Spheroidal culture is an anchorage-independent culture that positively selects cells that resist anoikis, which is programmed cell death induced on loss of cell-to-matrix interactions [10, 11]. Cells resistant to anoikis have been shown to exhibit cancer stem cell-like properties in various cancers [12]. The resulting spheroids can be propagated for many passages to increasingly enrich cells with enhanced cancer stem cell-like properties [13].

Of the many molecular mechanisms that regulate tumorigenesis, post-transcriptional regulation by circular RNA (circRNA) is important it is at the apex that controls other downstream regulatory mechanisms [14]. CircRNAs are back-splicing products of the pre-mRNA of a host transcript; multiple isoforms of different combinations of exon and intron sequences may be derived from a single host transcript, targeting and sponging off another group of post-transcriptional regulators, the microRNA (miRNA) [14–16]. CircRNA also functions via interacting with RNA-binding proteins, or in being translated into functional proteins to impact on downstream biochemical and cellular pathways [14, 16, 17]. Dysregulation of circRNAs in cancer has been associated with the enhancement of various cancer hallmarks [18].

In our previous expression profiling analysis of circRNAs in spheroids derived from two CRC cell lines, HCT-15 and WiDr, hsa_circ_0082096 was identified as one of top four up-regulated circRNAs in the spheroids compared with the parental CRC cells; bioinformatics analysis further predicts association of hsa_circ_0082096 with stem-cell properties [19]. The present work aimed to experimentally validate and to elucidate the role of hsa_circ_0082096 in modulating cancer stem cell properties and in the tumorigenesis process of colorectal cancer.

## Materials and methods

### Cell and spheroidal cultures and CRC patient tissues

All colorectal cancer (CRC) cell lines were obtained from American Type Culture Collection and cultured as suggested. The cell lines were regularly monitored for mycoplasma contamination. CRC spheroids were generated as previously described [19]. Primary CRC and adjacent normal paired tissues were obtained with consents from patients who underwent colectomy in Taipei Veterans General Hospital, Taiwan, between 2019–2022. in accordance with the International Ethical Guidelines for Biomedical Research Involving Human Subjects and was approved by the Medical Ethical Committee of Taipei Veterans General Hospital, Taiwan (IRB-2019–08-009B). The clinical CRC samples (*n* = 30) included 22 cases of patients with age ≥ 60 years old and 8 cases of age < 60, and 17 male and 13 female patients.

### RNA extraction, quantitative RT-PCR and sequencing analysis

RNA extraction using TRIzol reagent (Invitrogen, Thermo Fisher Scientific, USA), cDNA synthesis using SuperScript® III Reverse Transcriptase Kit (Invitrogen), quantitative reverse transcription-PCR (qRT-PCR) using SYBR® Select Master Mix (Invitrogen) using glyceraldehyde-3-phosphate dehydrogenase (GAPDH) as an internal control, and Sanger sequencing were as described [19]. The primers used are shown in Suppl. Table S1A.

### RNA pulldown and stem-loop qRT-PCR

The RNA pulldown protocol was modified from a previous work [20]. CRC cell lysates were collected using radio-immunoprecipitation assay buffer (RIPA) buffer and quantified. An aliquot of 2,000 µg cell lysate was incubated with 500 ng biotinylated circZNF800, or a scrambled control RNA. The mixture was gently agitated at 4 ºC for 4 h. Streptavidin Mag Sepharose beads (Cytiva, Massachusetts, USA) were incubated with the lysate overnight. After three washes using the bead washing buffer supplemented with RNase inhibitor provided by the vendor, the beads were resuspended in TRIzol and RNA was extracted and quantified by stem-loop qRT-PCR [21]. RNA (1 µg) was reverse transcribed with the stem-loop primers (Suppl. Table S1B) and qRT-PCR was performed as described in “RNA extraction, quantitative RT-PCR and sequencing analysis” section above, using U6 as the PCR control. Relative miRNA expression levels were calculated using the comparative C_т_ (ΔΔC_т_).

### Actinomycin D and RNase R treatments

CRC cells seeded at 5 × 10^5^ cells/well in a 6-well plate were incubated overnight. After treatment with 2.5 µg/mL of actinomycin D (Sigma-Aldrich, Merck, Germany), the cells were harvested for RNA extraction. For RNase R treatment, RNA extracted were treated with RNase R (Lucigen, Thermo Fisher Scientific, USA) based on the manufacturer’s instructions. The RNA was pelleted using lithium chloride precipitation (Thermo Fisher Scientific) and was resuspended in RNase-free water for further analysis.

### CircZNF800 synthesis in vitro

CircZNF800 synthesis in vitro was done using a permutated group I self-catalytic of *td* (thymidylate synthase) gene intron plasmid, driven by *T7* RNA polymerase, a gift from Professor Manuel Ares [22]. The circZNF800 cDNA, or the green fluorescence protein (GFP), sequence was cloned at the AflII and MfeI restriction sites between the 3’ and 5’ splice sites of the plasmid. In vitro transcription was performed using the HiScribe™ T7 Quick High Yield RNA Synthesis Kit, (New England Biolabs, Ipswich, MA, USA) per manufacturer’s instructions. The resulting RNA was purified by lithium chloride precipitation. For modified RNA, cyanine-3-uridine-5'-triphosphate (Cy3-UTP) was added at a 1:4 ratio. RNA circularization was induced by first heating the RNA to 65 ºC for 3 min and placed on the ice for 10 min. The RNA was mixed with guanosine-5'-triphosphate (GTP) and MgCl_2_ at a final concentration of 10 mM in 1 × polynucleotide kinase reaction buffer (PNK) buffer (New England Biolabs). The mixture was heated at 55 ºC for 35 min. The RNA was purified using lithium chloride precipitation and resuspended in nuclease-free water for analysis in an agarose-formaldehyde gel (2%) in 3-(N-morpholino) propanesulfonic acid (MOPS) buffer and deionized. The agarose gel was visualized under UV light.

### Preparation of CRISPR-Cas13d circZNF800 constructs

The CRISPR Cas13d plasmid was purchased from Addgene (#138148, Massachusetts, USA). Two CRISPR RNA sequences, crRNA1 and crRNA2, were designed and synthesized based on the backsplice junction of circZNF800 and were cloned into the BsmBI restriction site of the plasmid. The constructs were sequence verified. A control construct, crSC, which harbored a scrambled sequence, was also prepared.

### DNA and RNA transfection

CRC cells were seeded at 5 × 10^5^ cells/well in a 6-well plate and incubated overnight. The DNA or RNA transfection was performed using Lipofectamine 2000 (Invitrogen) per manufacturer’s instructions.

### RNA FISH and dual immunofluorescent assay

A cyanine-5 (Cy5)-tagged circZNF800 antisense RNA was commercially synthesized (MDBio Inc, Taiwan), and was mixed with the hybridization buffer at a final concentration of 200 nM in an overnight incubation. RNA FISH protocol was modified from previous works [23]. After hybridization and washing (see Suppl. Table S1C for probe information), the slides were stained with 4’,6-diamidino-2-phenylindole (DAPI) (Invitrogen, Thermo Fisher) at 1:10,000 dilutions in phosphate buffered saline (PBS) for 10 min. Fluorescent images were captured using a FV3000 Confocal Laser Scanning Microscope (Olympus, Japan). For dual RNA-FISH and protein immunofluorescent staining, upon overnight incubation with the circRNA probe as described above, the tissue slides were rinsed with saline sodium citrate (SCC) buffer before incubation with antibodies for Lgr5 (ab219107, Abcam, UK), Sox9 (14–9765-82, eBioscience, Thermo Fisher Scientific) or Ki-67 (#9129, Cell Signalling Technology, MA, USA). Goat anti-mouse IgG secondary antibody conjugated with Alexa Fluor 546 and goat anti-rabbit IgG secondary antibody conjugated with Alexa Fluor 488 (Invitrogen) were used. The fluorescence images were captured as described above.

### Flowcytometric analysis of protein markers and cell proliferation rate

CRC cells were harvested using Accutase (Gibco, Thermo Fisher) in 5 × 10^5^ cells aliquots. The antibodies used were CD44 (#3570, Cell Signalling Technology), CD133 (#5860, Cell Signalling Technology), Lgr5 and SOX9. Cell proliferation rate was assessed using a commercial EdU Staining Proliferation Kit (fluor 488) (Abcam, UK) per manufacturer’s instructions. The treated cells were collected through centrifugation and washed with once with Permeabilization Buffer. The cell pellet was resuspended in 300 µl PBS and data acquisition was achieved by flowcytometry as described above.

### Colony forming assay

CRISPR Cas13d-transfected CRC cells were seeded at 2.5 × 10^3^ cells in a 6-well plate. The cells were cultured in the respective media and replenished every other day. The cells were allowed to form colonies for 10 – 14 days. Crystal violet staining for 20 min at room temperature was used to visualize the colonies formed.

### Analysis of the effects of circZNF800 over-expression and knockdown in mice

BALB/c nude male mice (strain CAnN.Cg-Foxn1^nu^/CrlBltW) were purchased from Bio-LASCO Taiwan, Taiwan, and were housed in cages with free access to food source and water. The animal work was conducted according to the Guidelines for Laboratory Animals in the Taipei Veterans General Hospital, with the institutional ethical review board approval (IACUC 2012–079).

To evaluate the effects of circZNF800 over-expression on tumor growth, HCT-15 and WiDr cells in PBS were first injected subcutaneously at 2.5 × 10^6^ cells per dorsal flank of nude mice until tumors were formed to an average size of ~ 500 mm^3^. The mice were divided into the vehicle (saline), circGFP RNA- and circZNF800-treated groups (*n* = 3 per group). RNA (10 µg) in TransIT®-QR Delivery Solution (Mirus Bio, Wisconsin, USA), or 10 µL saline, was diluted in 90 µL of TransIT®-QR Delivery Solution to a final volume of 100 µL. The mixture was injected nto the tumor sites within 15 min of preparation (*n* = 3). The treatment was performed 4 times over 10 days, after which the tumors were left to grow for 20 days, and tumor sizes were measured every 4 days using a caliper. The mice were sacrificed and the tumors were harvested for imaging.

To analyze the effects of circZNF800 knockdown in mice, circZNF800 was stably knocked down in HCT-15 and WiDr cells by lentivirus transduction of CRISPR-Cas13d circZNF800 knockdown constructs crRNA1 and crRNA2 and the control crSC. The cells were suspended in PBS for injection at 2.5 × 10^6^ cells per flank of nude mice. The tumors were left to grow for 24–30 days while tumor sizes were monitored every 3–5 days.

### Analysis of the effects of intratumoral circZNF800 knockdown in mice

For intratumoral circZNF800 knockdown, purified and quantified lentiviruses wereobtained from the RNAi Core, Academia Sinica, Taiwan. Each flank was administered 1 × 10^8^ lentivirus particles carrying the control crSC or the targeting crRNA1 or crRNA2 (*n* = 2). The treatment was performed 4 times over 10 days. The tumors were left to grow for 16–22 days in WiDr and HCT-15 cells, respectively, due to different growth rates. Tumor sizes were monitored before the tumors were harvested for imaging.

### Bioinformatics analysis

Messenger RNA sequences were derived from GenBank, NCBI; https://www.ncbi.nlm.nih.gov/genbank/); circRNA sequences and isoforms were derived from circBase (http://www.circbase.org) and circBank (http://www.circbank.cn); miRNA sequences were derived from miRBase (https://www.mirbase.org). CircRNA-miRNA interactions were analyzed using the CircInteractome database (https://circinteractome.nia.nih.gov). MiRNA-mRNA interactions were analyzed by using the TargetScanHuman 7.0 database (https://www.targetscan.org/vert_70/). All bioinformatics analyses using the above databases were done in January–February 2023.

### Statistical analysis

All experiments were performed in triplicates. The triplicated data were evaluated for statistical significance using the standard paired Student’s *t*-test. Comparisons were made between the control group and the treated group, unless specified otherwise. The Prism GraphPad software was used to evaluate the significance levels and for graph construction.

## Results

### CircZNF800 expression is up-regulated in in CRC-derived spheroids and CRC tumor tissues

**Fig. 1:**
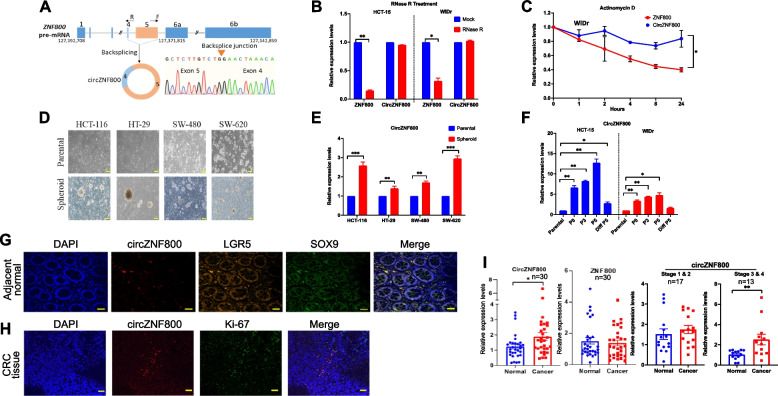
CircZNF800 expression is up-regulated in in CRC-derived spheroids and CRC tumor tissues. **A** Schematic illustration of circZNF800 biogenesis via backsplicing of exons 4 and 5 of the *ZNF800* pre-mRNA (NC_000007.14). Two *ZNF800* alternative transcripts carry either exon 6a (NM_176814.5) or 6b (XM_011515859.4) are shown. F and R indicate the forward and reverse primers used in sequencing of the backsplicing junction. **B**, **C** Stability analysis of circZNF800 in WiDr cells on RNase R (**B**) and actinomycin D (**C**) treatments. The relative RNA levels were determined by qRT-PCR. *P < 0.05 and **P < 0.01 relative to those of the untreated sample (**B**) or the *ZNF800* transcript (**C**). **D** Morphology of the CRC-derived spheroids. **E** Up-regulated circZNF800 expression in spheroids derived from CRC cell lines. **F** Passage (P)-dependent up-regulated circZNF800 expression in CRC cell-derived spheroids and on serum-induced differentiation of the spheroids at passage 5 (Diff. P5). **G**, **H** Co-localization of circZNF800 and intestinal stem cell markers LGR5 and SOX9 in the adjacent normal tissue (**G**) or Ki-67 high proliferative marker (**H**) in a CRC tumor tissue in dual RNA FISH-immunofluorescent assays. Bars represent 100 µm. **I** Significant circZNF800 over-expression in paired tumor and normal tissues of CRC patients, and in late-stage CRC tumors. Paired tissue samples (*n* = 30) were subjected to qRT-PCR analysis to determine the circZNF800 and ZNF800 expression levels (left two panels); the circZNF800 expression data from the same patient cohort were further grouped according to pathological stages 1 & 2 (*n* = 17) and 3 & 4 (*n* = 13) (right two panels) for circZNF800 expression analysis. *p < 0.05, **p < 0.01 and ***P < 0.001 relative to the control samples as indicated or the adjacent normal tissues

In our previous study, we have identified hsa_circ_0082096 as one of top four up-regulated circRNAs in spheroids derived from the HCT-15 and WiDr CRC cell lines [19]. Based on circBase and circBank databases, the host transcript of hsa_circ_0082096 is *ZNF800* (zinc-finger protein 800). The *ZNF800* gene (NM_176814 and XM_01151585.4) is composed of seven exons, with an alternative exon 6a or 6b, and is predicted to generate eight circRNA isoforms, including hsa_circ_0082096, based on expression profiling studies (Suppl. Table S2) [19, 24–26]. The hsa_circ_0082096 isoform that we reported earlier is derived from the back-splicing of exons 4 & 5 of the *ZNF800* transcript 1,837 nucleotides in length and is designated as hsa_circZNF800, or circZNF800 for short (Fig. 1A). It is noted in the various expression profiling reports that circZNF800 is also expressed in the chronic myeloid leukemia cells K562 and in SK-N-SH-RA, a neuroblastoma SK-N-SH cells differentiated with retinoic acid, and in normal fetal lung fibroblast AG04450 cells, EBV-transformed lymphoblastoid cells, chondrocytes and in multiple brain tissues and neuronal cells (Suppl. Table S2). Taken together, expression profiling reports, including our previous work, have predicted participation of circZNF800 in tumorigenesis, cancer stem cells and the functioning of brain tissues. CircZNF800 in CRC cells was confirmed by RNase R resistance (Fig. 1B) and significantly longer half-life than the host transcript *ZNF800* on actinomycin D challenge (Fig. 1C) in the two CRC cell lines tested.

Since our previous report on circZNF800 in two CRC cell lines [19], we confirmed here that circZNF800 expression was consistently up-regulated in spheroids generated from a panel of four other CRC cell lines (Fig. 1D and E). Furthermore, circZNF800 up-regulation was passage-dependent, and was down-regulated on serum-induced differentiation in passage 5 cells (Fig. 1F), supporting involvement of circZNF800 in modulating CRC stem cell properties in maintaining the spheroidal phenotype. The cancer stem cell population in CRC is thought to have arisen from a mutated ISC population [5], and that LGR5 and SOX9 are ISC markers that reside in the crypt base columnar cells of the tissue [27]. Dual immunofluorescence assays showed that circZNF800 co-resided in the LGR5^high^/SOX9^high^ cell population in non-pathological colorectal tissues (Fig. 1G), supporting association of circZNF800 with ISC. Dual immunofluorescence assays further showed co-location of circZNF800 and the Ki-67 high proliferation marker in the same tumor cell population (Fig. 1H) [28]. In clinical biopsies, circZNF800 expression, determined by qRT-PCR, was significantly higher in CRC tumor tissues than in the paired adjacent normal tissues analyzed (*n* = 30), whereas the *ZNF800* transcript levels were not significantly altered between the tumor and normal tissues (Fig. 1I, left two panels). However, when the circZNF800 expression data were further grouped according to pathological stages 1 & 2 (*n* = 17) and 3 & 4 (*n* = 13), significant up-regulated circZNF800 expression was found in the late stages 3/4 tumor tissues, but not in the early stages 1/2 samples (Fig. 1I; right two panels). Taken together, the data show that up-regulated circZNF800 expression is associated with cancer stem cell properties and CRC tumorigenesis.

### Strategies for circZNF800 over-expression (OE) and knockdown (KD) in cells

**Fig. 2:**
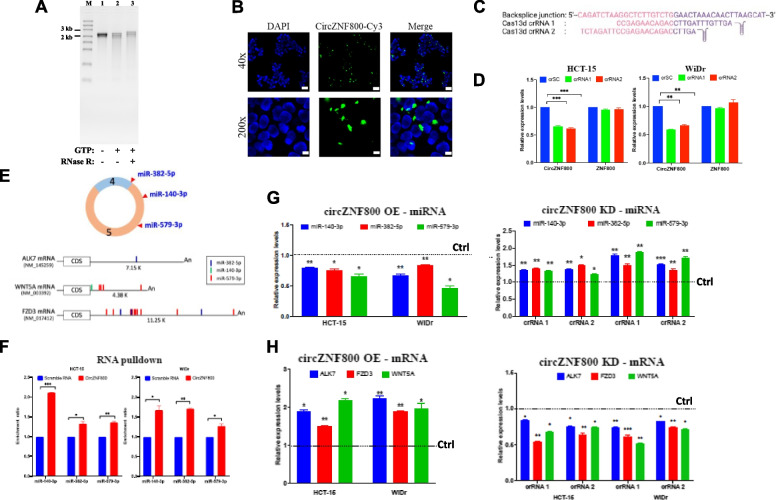
CircZNF800 sponges miR-140-3p, miR-382-5p & miR-579-3p to up-regulate expression of *ALK7, FZD3 & WNT5A*. **A**, **B** Properties of in vitro transcribed and circularized circZNF800: RNase R resistance shown in a 2% formaldehyde-agarose gel (**A**) and co-localization of circZNF800-Cy3 RNA transfected in WiDr cells observed under a fluorescence microscope (**B**). **C**, **D** For CRISPR-Cas13d knockdown of circZNF800, the two CRISPR RNA (crRNA) constructs, crRNA1 and crRNA2 (**C**), and the knockdown efficiency (**D**) are shown. In (**D**), CrSC was a crRNA construct with scrambled sequence used as a reference control. Relative expression levels of circZNF800 and *ZNF800* post-crRNA transfection were determined by qRT-PCR, normalizing to the levels of the control crSC-transfected cells. **p < 0.01 and ***p < 0.001 were values relative to the CrSC cells. **E** Mapping of the miRNA seed sequence-interacting sites on circZNF800 (top panel) and the 3’-UTR sequences of the targeted transcripts (bottom panel) (see Suppl. Figure S1 for sequence alignment details). The relative positions of the miRNAs, the GenBank accession numbers of the mRNA sequences and the 3’-UTR lengths are shown. CDS, coding sequence; An, polyA tail. **F** RNA pulldown assays using biotinylated circZNF800 in HCT-15 and WiDr cells. The pulled-down RNA products were quantified in stem loop qRT-PCR (see Suppl. Table S1 for primer details) using a scramble RNA as a control and normalized to 1.0. **G**, **H** CircZNF800 over-expression (OE) sponges while knockdown (KD) up-regulates expression of the targeted miRNAs (**G**) and mRNAs (**H**) in CRC cells relative to the controls without OE and KD transfection, normalized to 1.0 as shown by the horizontal control (Ctrl) dashed lines. OE and KD were achieved by transfection of the in vitro synthesized circZNF800 and the CRIPSR constructs, respectively, in CRC cells for 48 h before RNA quantification by stem loop qRT-PCR relative to the control values (horizontal Ctrl dashed lines). *P < 0.05, **P < 0.01 and ***P < 0.001 were values relative to the specified controls

To further dissect molecular and cellular events affected by circZNF800, experimental approaches to manipulate cellular levels of circZNF800 were developed. For over-expression (OE), an in vitro scheme of generating self-circularizing circular RNA using permutated T4 phage *Td* gene was used [22, 29]. The RNA products obtained were ~ 1,800 nucleotides as expected and were RNase R resistant supporting circularity (Fig. 2A; see also Suppl. Fig. S1). The in vitro transcribed and circularized circZNF800 was confirmed to carry exons 4 and 5 sequences by sequencing. To assess cellular uptake, a modified UTP-conjugated circZNF800 RNA tagged with fluorescence Cy3 was transfected in WiDr cells and was visualized by fluorescence microscopy 12 h post-transfection. The fluorescence images showed that the tagged circZNF800 overlapped with the WiDr cells (Fig. 2B), suggesting successful circZNF800 uptake by the cells.

For circZF800 knockdown (KD), the CRISPR-Cas13d system, which induces specific circRNA degradation, was used [30, 31]. Two CRISPR RNA (crRNA) constructs, crRNA1 and crRNA2, that span different sequences of the circZNF800 back-splice junction, were designed (Fig. 2C). A similar construct with a scrambled sequence, designated crSC, was used as a control. Both crRNAs were able to induce ~ 40% significant knockdown of circZNF800 in two CRC cell lines with little effects on the *ZNF800* transcript compared with the crSC control-treated cells (Fig. 2D).

### CircZNF800 sponges miR-140-3p, miR-382-5p and miR-579-3p and up-regulates expression of *ALK7/ACR1C*, *FDZ3* and *WNT5A*

In our previous expression profiling analysis, miR-140-3p, miR-382-5p and miR-579-3p were predicted to be sponged by circZNF800 to collectively affect expression of the *ALK7/ACR1C* (activin A receptor type 1C)*, FDZ3* (frizzled class receptor 3) and *WNT5A* (WNT family member 5A) genes in spheroids derived from CRC cell lines [19]. The predicted circZNF800-miRNA-mRNA interactions are supported by sequence alignments of the seed sequences of the circZNF800-miRNA and miRNA-mRNA pairs using the CircInteractome data (Fig. 2E). While miR-382-5p is mapped in the short exon 4, the other two miRNAs are mapped in exon 6 of the *ZNF800* transcript (Fig. 2E, top panel; Suppl. Fig. S2A for sequence alignment details). On miRNA-mRNA alignments using the TargetScan database, the 3’-untranslated region (3’-UTR) of the 7.15-kb *ALK7* transcript is found to be targeted specifically by the seed sequence of miR-382-5p, while the 4.38-kb 3’-UTR sequence of *WNT5A* is targeted by the seed sequences of miR-140-3p once and miR-579-3p at four sites (Fig. 2E). Interestingly, the 11.25-kb 3’-UTR of the *FZD3* transcript is targeted three times by miR-382-5p and ten times by miR-579-3p, which are clustered in the 5’-half of the UTR sequence (Fig. 2E). The seed sequences of the miRNA-mRNA interactions are 7–8 nucleotides in length and in perfect sequence homology, supporting with high confidence the bioinformatics-predicted miRNA-mRNA interactions (Suppl. Fig. S2B). To validate the predicted circZNF800-miRNA interactions, biotinylated-circZNF800 and a scrambled RNA control generated in transcription in vitro were used in RNA pulldown assays in HCT-15 and WiDr cells. The pulled-down RNA products were purified and subjected to stem-loop qRT-PCR analysis. The results indicated that the circZNF800-pulldown products were enriched in the predicted miRNAs in the HCT-15 and WiDr cells compared to the scrambled RNA control (Fig. 2F).

Using the over-expression and knockdown methodologies described in Fig. 2A-D above, ectopic circZNF800 OE in CRC cells was shown to result in significant down-regulation of the expression levels of all three miRNAs while circZNF800 KD up-regulated the expression of the miRNAs compared to the control (Fig. 2G), collaborating with circZNF800 sponging of the miRNAs. Furthermore, circZNF800 OE significantly up-regulated the mRNA levels of *ALK7*, *FZD3* and *WNT5A* while the reverse was observed on circZNF800 KD (Fig. 2H), supporting circZNF800-miRNA and miRNA-mRNA interactions (Fig. 2E). The data collectively show that by sponging miR-140-3p, miR-382-5p and miR-579-3p, circZNF800 promotes *ALK7, FZD3* and *WNT5A* expression, possibly contributing to cancer stem cell properties of CRC cells.

### CircZNF800 up-regulates CSC markers in CRC cells

**Fig. 3:**
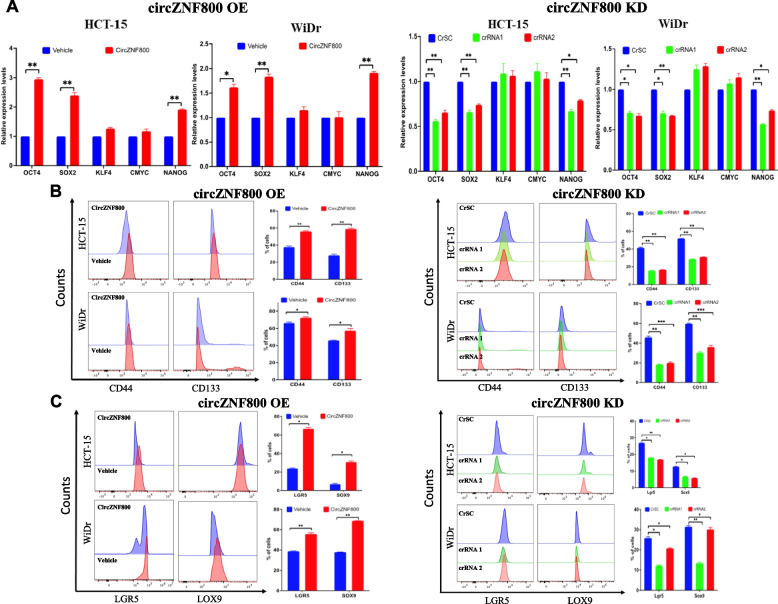
CircZNF800 up-regulates expression of CSC markers in CRC cells. Effects of circZNF800 over-expression (OE) (left panels) and knockdown (KD) (right panels) on the expression levels of the pluripotency markers determined by qRT-PCR (**A**), the CSC markers CD44 and CD133 (**B**) and the intestinal stem cell markers LGR5 and SOX9 determined by flowcytometry (**C**). Representative flowcytometry histograms are shown. Half-offset plots are used to illustrate the shifts of the markers. Scales of 1–0 units used in all cases were algorithmically determined by FlowJo software. Bar charts represent triplicated quantitative analysis. *p < 0.05 and **p < 0.01 were values relative to the scrambled CrSC or vehicle control group

The pluripotency markers NANOG and the Yamanaka factors OCT4, SOX2, KLF4 and c-MYC have previously been shown to be linked with enhanced cancer stem cell properties in cancer cells [32–34]. On circZNF800 over-expression, *OCT4, SOX2* and *NANGO* were significantly up-regulated in the two CRC cell lines tested in qRT-PCR analysis; on circZNF800 knockdown, the same three pluripotency factors were also down-regulated (Fig. 3A). The data support circZNF800-mediated modulation of expression of the core regulators, OCT4, SOX2 and NANOG, in maintaining stemness properties in the CRC population. The impacts of circZNF800 OE and KD on the expression levels of the CSC markers, CD133 and CD44, and the intestinal stem cell (ISC) markers, LGR5 and SOX9 (see above) were also investigated by flowcytometry (Fig. 3B and C). CD133^+^ cells isolated from CRC cell populations have been shown to mimic properties of CRC cancer stem cells and CD44^+^ cells exhibit enhanced tumorigenicity and cell proliferation in CRC [35, 36]. The results showed that the expression levels of CD133 and CD44 were up-regulated on circZNF800 OE, and down-regulated on circZNF800 KD (Fig. 3B). The expression levels of LGR5 and SOX9 were also up- or down-regulated on circZNF800 OE and KD, respectively (Fig. 3C), echoing the previous observation that LRG5 and SOX9 are enriched in the normal colorectal tissue (Fig. 1G). These results support circZNF800 regulation of the expression of these cancer stem cell markers in CRC cells.

### CircZNF800 promotes cell proliferation and sphere and colony formation in CRC cells in vitro

**Fig. 4:**
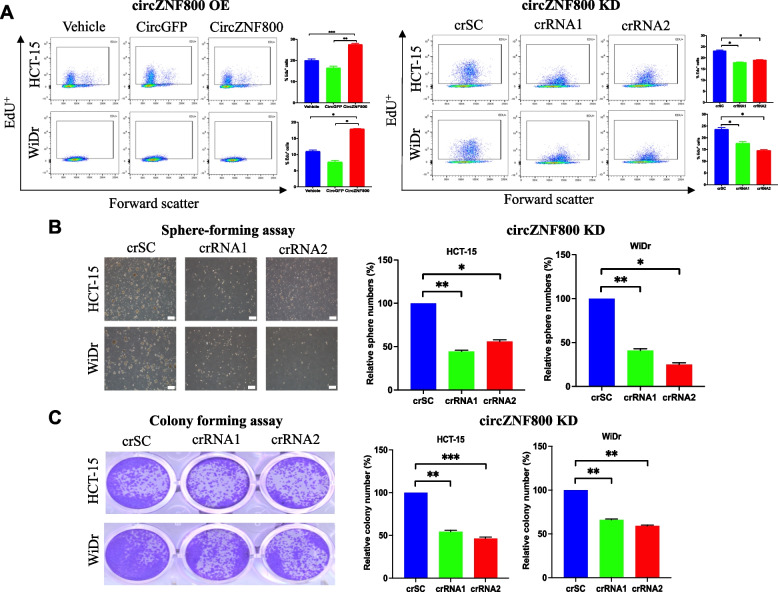
circZNF800 promotes cell proliferation and sphere and colony formation in CRC cells in vitro*.* **A** Effects of circZNF800 on cell proliferation. On circZNF800 over-expression (OE) (left panels) or knockdown (KD) (right panels), the cells were treated with EdU and analyzed by flowcytometry. Representative flowcytometry histographs are shown. Bar charts represent triplicated quantitative analysis. *P < 0.05, **P < 0.01, ***P < 0.001 relative to the respective controls. **B** Spheroid formation on circZNF800 knockdown. Images were taken after 10 days in the spheroid culture conditions; spheroid quantification is shown (bar represents 100 µm). Colony quantification is also shown. **C** Colony formation of CRC cells on circZNF800 knockdown. Images show the resulting colonies on staining with a crystal violet solution

The cellular effects of circZNF800 over-expression and knockdown on other cancer stem cell properties were next investigated. The effects on cell proliferation were evaluated by EdU assay and flowcytometric analysis. The results showed increased EdU-positive cell population on circZNF800 over-expression compared to the vehicle and the mock circularized green fluorescence protein (GFP) RNA, circGFP; the data were supported by a reverse effect on knocking down circZNF800 (Fig. 4A). The EdU assay data indicated circZNF800-mediated promotion of cell proliferation, in line with the clinical findings that circZNF800 was up-regulated in CRC tumor samples (Fig. 1I). Furthermore, circZNF800 knockdown in CRC cells led to impeded spheroid and colony forming abilities (Fig. 4B and C), supporting that silencing circZNF800 suppresses cancer stem cell phenotype and cellular transformation in vitro. It was also noted that the spheroids formed on circZNF800 knockdown were smaller and irregular in morphology, indicating compromised cellular processes. These data collectively demonstrate that circZNF800 modulates cellular processes pertaining to cancer stem cell properties, leading to enhanced tumor growth in vitro. Due to the high toxicity observed in circZNF800 over-expression cells on extended period of culture before data collection, the colony and spheroid formation assays were not performed on circZNF800 ectopic over-expression cells.

### CircZNF800 over-expression promotes while knockdown suppresses tumor growth in vivo

**Fig. 5:**
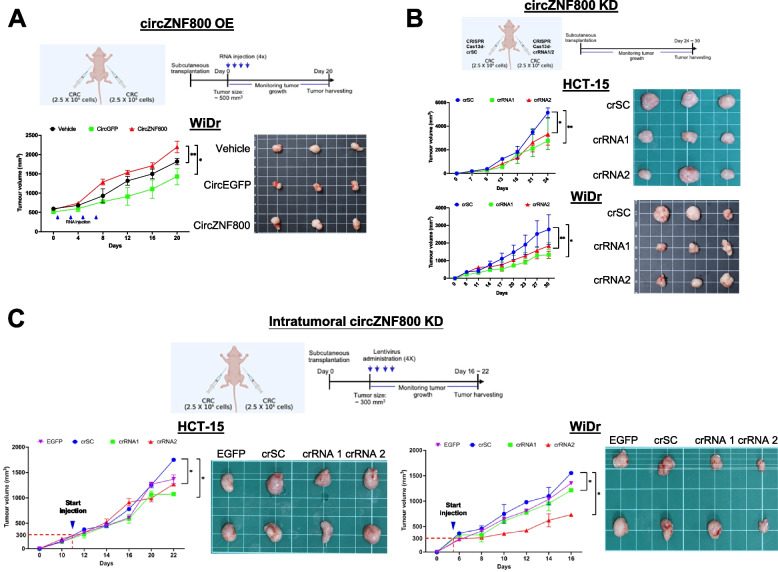
CircZNF800 over-expression promotes while knockdown suppresses tumor growth in vivo. **A** CircZNF800 over-expression promotes tumor growth in vivo. WiDr cells were first transplanted subcutaneously into nude mice. The mice were divided into three groups: the vehicle group (saline) and the two groups injected either with circZNF800 RNA or circEGFP RNA (*n* = 3 for each group). When the tumors reached ~ 500 mm^3^, each group was injected four times over 10 days with 10 µg RNA for each group. Tumors were harvested on day 20 from the start of the RNA injection (schematic panel). Tumor growth rates and images of the harvested subcutaneous CRC tumors on day 20 of treatment are shown. **B** CircZNF800 knockdown suppresses tumor growth in vivo. Stably knockdown circZNF800 CRC cells were subcutaneously transplanted in nude mice and the tumors were harvested on day 24 (HCT-15 cells) or on day 30 (WiDr cells) post-cell injections (*n* = 3). Tumor growth rates and images of the harvested subcutaneous CRC tumors are shown. **C** Intratumoral administration of the circZNF800-knockdown lentiviral CRISPR-Cas13d constructs retards tumor growth in mice. HCT-15 or WiDr cells were subcutaneously transplanted in nude mice and the tumors were allowed to grow to an average size of ~ 300 mm^3^. Purified lentivirus constructs of either the control crSC, or crRNA1 or crRNA2 were injected at the tumor sites twice a week for 10 days. The tumors were allowed to continue to grow up to 16 or 22 days for WiDr or HCT-15, respectively, before harvesting for analysis (*n* = 2). *p < 0.05 and **p < 0.01 were relative to the crSC or vehicle-treated group

The effects of circZNF800-mediated effects observed in vitro were further assessed in vivo in xenograft mice (Fig. 5). For over-expression analysis, WiDr cells were first transplanted subcutaneously to nude mice, and the tumors were allowed to grow to ~ 500 mm^3^ before injecting the in vitro synthesized circZNF800 RNA at the tumor sites four times over 7 days. Growth of the tumor were monitored up to 20 days starting from the first RNA injection (Fig. 5A, top panel). CircZNF800-treated mice showed significant enhanced tumor growth rate and developed larger tumors compared to the two control groups injected with saline (vehicle group) or circularized EGPF (Fig. 5A). The data echo the in vitro data that circZNF800 promotes cellular proliferation (Fig. 4A).

The effects of circZNF800 knockdown on tumor growth were assessed in two different experimental approaches. In the first approach, stable circZNF800-knockdown HCT-15 and WiDr cells were first generated using the CRISPR Cas 13d-based crRNA1 or crRNA2, and crSC negative control carrying a scrambled sequence; subsequently, the KD CRC cells were subcutaneously transplanted in nude mice, and the tumor growth was monitored for up to 24 days for HCT-15 or 30 days for WiDr cells due to different growth rates of the two cell lines (Fig. 5B, top panel). In both cell lines, tumor growth on the crRNA1- and crRNA2-treated cells was significantly slower than that in the scrambled control crSC cells (Fig. 5B).

In the second approach, the effects of circZNF800 knockdown in tumors in nude mice developed from subcutaneously grafted CRC cells were investigated. When the tumor grew to ~ 300 mm^3^, each tumor site was injected four times each with 1 × 10^8^ purified lentiviral particles harboring crRNA1 or crRNA2, or the EGFP or crSC control constructs (Fig. 5C, top panel). Due to the different growth rates, the HCT-15 and WiDr-injected mice were monitored for 22 or 16 days, respectively, by measuring the tumor size regularly before the mice were sacrificed. Using the crSC as a control reference, tumor growth in the crRNA1- and crRNA2-treated mice was significantly retarded (Fig. 5C). Despite the small number of mice treated with each crRNA (*n* = 2 each), two KD constructs were used and each in two different cell lines (total *n* = 8), the data collectively support that intratumoral circZNF800 knockdown leads to suppressed tumor growth in the mice. The in vivo data obtained are in line with the in vitro data that circZFN800-knockdown suppresses cell proliferation, colony and spheroid formation (Fig. 4B-C). The data may be a first hint to indicate the possibility of delivering crRNA1- and crRNA2-like viral constructs for therapeutic treatment of colorectal cancer in humans.

## Discussion

**Fig. 6:**
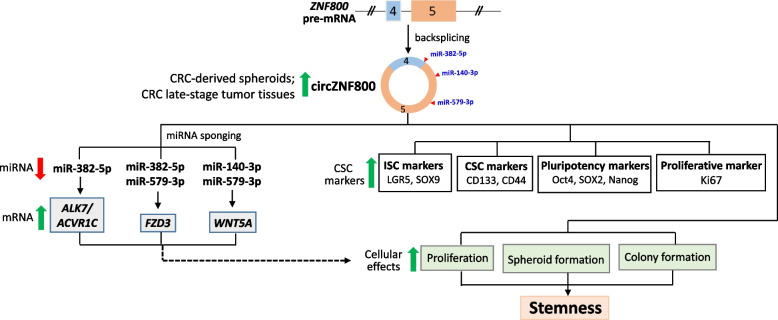
Schematic summary of the predicted role of circZNF800 in regulating cancer stem cell properties via multiple miRNA-mRNA axes in CRC tumorigenesis. The up- (green) and downward (red)-pointing thick arrows indicate up- and down-regulated expression, respectively, of the indicated parameters demonstrated in this work. Briefly, circZNF800 in late-stage CRC or CRC-derived spheroidal cells suppresses miR-140-3p, miR-382-5p and miR-579-3p to up-regulate expression of *ALK7, FZD3* and *WNT5A* (in grey boxes). On the other hand, circZNF800 up-regulates the expression of cancer stem cell markers (in white boxes) and various cellular processes (green boxes) leading to enhanced stemness properties of CRC cells in the tumorigenesis process. The dashed-line arrow indicates the predicted connection of the circZNF800-miRNA-mRNA axes with the affected downstream cellular events

Expression of circZNF800 is previously shown to be up-regulated in CRC-derived spheroids [19]. We show here that circZNF800 is up-regulated in expression in late-stage CRC tumors (Fig. 1I), which may be enriched in cancer stem cells, consistent with circZNF800 over-expression in the spheroids. By manipulating the cellular levels of circZNF800 via over-expressing or knockdown of the circRNA (Fig. 2A-D), circZNF800 was shown to suppress expression of miR-140-3p, miR-382-5p and miR-579-3p and to up-regulate expression of the *ALK7/ACVR1C, FZD3* and *WNT5A*, as predicted by bioinformatics analysis (Fig. 2E-H). CircZNF800 was further shown in in vitro experiments to positively modulate the expression of pluripotency and cancer stem cell markers (Fig. 3). Functionally, the circRNA promoted cell proliferation, spheroid and colony formation (Fig. 4). The in vitro data are supported by in vivo experiments in which ectopic circZNF800 over-expression led to accelerated while circZNF800 knockdown retarded tumor growth in xenograft mice (Fig. 5). The data collectively support the hypothesis that circZNF800 plays a major role in the CRC tumorigenesis process via modulating cancer stem cell properties, as summarized in Fig. 6.

Dysregulated expression of circRNA has been implicated in the tumorigenesis of various types of cancer [37]. Data presented in this study indicate that over-expression circZNF800 contributes to CRC tumorigenesis via exerting its detrimental regulatory effects in the intestinal stem cells and the high proliferative cell population (Fig. 1G and H), consistent with circZNF800-mediated enhancement of cancer stem cell-like properties in CRC. Involvement of other circRNAs in mediating stemness has been reported in other cell types, including induced pluripotent and cancer stem cells [38]. Over-expression of circBIRC6 promotes and maintains the pluripotency state of human embryonic stem cells by regulating the expression levels of *OCT4* and *NANGO* [39]. Likewise, circNOTCH1 over-expression in gastric cancer stem cells is linked with maintenance of stemness [40]. In bladder cancer, however, circGPRC5A is shown to be translated into a GPRC5A protein to regulate the bladder cancer stem cell phenotype in promoting tumorigenicity [41]. On the other hand, the host transcript *ZNF800* of circZNF800 has been linked to regulation of apoptosis, migration and invasion of lung cancer cells, and acts as an adipogenesis master regulator associated with cardio-metabolic traits [42, 43].

The three miRNAs sponged by circZNF800 and the three miRNA-targeted mRNAs have been reported to modulate cancer stem cell-related properties, including cellular proliferation and hence, tumor progression, migration and invasion in metastasis, relapses and chemosensitivity in other cancer types, as summarized in Table 1. Besides circZNF800, only miR-382-5p is also reported to be sponged by another circular RNA circUBAP2 to regulate *GPRC5A* (G protein-coupled receptor class C group 5 member A) expression in glioma [44]. Moreover, miR-382-5p is also most versatile in also targeting four other mRNAs, *YB-1, ANXA3, KLF12* and *HIPK3*, besides *GPR5A* (Table 1) [45–47]. In the targeted mRNA group, the most prevalent transcript associated with effects on cancer stemness is WNT5A that acts via the WNT/β-catenin signaling pathway (Table 1) [48–52]. Collectively, the circZNF800-regulated miRNAs and mRNAs reported in various cancer types affect cancer cell stem properties via the WNT/β-catenin, PI3K/AKT and TGFβ-SMAD signaling pathways, amongst others (Table 1) [44–57]. In short, circZNF800 acts as a switch that negatively controls the function of a specific group of downstream miRNAs, which, in turn, regulate expression of targeted downstream genes to promote cancer stemness, contributing to the many features of human cancers.

**Table 1:** Other circRNA-miRNA-mRNA axes and affected signaling pathway and cancer stemness reported for the circZNF800-regulated circRNAs and mRNAs

Cancer	circRNA/miRNA /mRNA^a^	Signaling pathway affected	Cancer stem cell-associated properties	Reference
**A. circZNF800-modulated miRNA group**				
Lung adenocarcinoma	**miR-140-3p**	WNT/β-catenin	Chemosensitivity	[53]
Glioma	CircUBAP2 / **miR-382-5p /** *GPRC5A*	NA	Cell proliferation, migration & invasion	[44]
Osteosarcoma	**miR-382-5p /** *YB-1*	NA	Metastasis & relapse	[47]
Pancreatic	**miR-382-5p /** *ANXA3*	PI3K/AKT	Tumor progression	[45]
Colorectal	**miR-382-5p /** *KLF12 & HIPK3*	NA	Cell proliferation, migration, invasion & chemosensitivity	[46]
Glioblastoma multiform	**miR-579-3p /** *AKT1*	PI3K/AKT	Cell proliferation & migration	[54]
**B. circZNF800-modulate mRNA group**				
Prolactinoma	***ALK7***	Activin	Cell proliferation	[55]
Retinoblastoma	***ALK7***	ALK7/SMAD2	Cell proliferation & invasion	[56]
CML	circCBFB / miR-607 / ***FZD3***	WNT/β-catenin	Cell proliferation	[57]
Gastric	***WNT5A***	NA	Cell migration &invasion	[49]
Erb2-induced breast cancer	***WNT5A***	TGFβ-SMAD	Expansion of tumor-initiating cells	[48]
NPC	***WNT5A***	Promoted EMT	Increased CD24-CD44 + cells & metastasis	[51]
NSCLC	CircVAPA / miR-876-5p / ***WNT5A***	WNT/β-catenin	Cell proliferation, migration, invasion and stemness	[50]
Bladder	miR-374a / ***WNT5A***	NA	Metastasis and invasiveness	[52]

*CML* chronic lymphocytic leukemia, *NPC* nasopharyngeal carcinoma, *NSCLC* non-small cell lung cancer, *GPRC5A* G protein-coupled receptor class C group 5 member A, *YB-1* Y box-binding protein 1, *ANXA3* Annexin A3, *KLF12* Krüppel-like factor 12, *HIPK3* homeodomain-interacting protein kinase 3, *AKT1* AKT serine/threonine kinase 1 *NA* not available

^a^The circZNF800-regulated miRNAs and mRNAs are shown in bold letters

We also demonstrated here that CRISPR Cas13d-mediated circZNF800 knockdown was highly specific and induces suppressed cell proliferation and colony formation in vitro and tumor growth in vivo in CRC cells, as has been reported for the silencing other circRNAs in other cancer types [30, 31, 58]. The high sequence specificity of the CRISPR Cas13d system is assured by the observation that a single mismatch in the CRISPR RNA-targeting sequence of a circRNA could significantly reduce the knockdown efficiency, thus, avoiding non-specific knockdown [30]. Integrating the CRISPR Cas13d-mediated circZNF800 knockdown system reported here with other more human compatible and effective delivery vectors, such as the adeno-associated virus, could be clinically feasible in intervention treatment of colorectal cancer [59].

## Conclusions

We show in this work that circZNF800 is over-expressed in late-stage colorectal tumors and in cancer stem cell-enriched spheroids derived from CRC cells. CircZNF800 is shown to sponge a small set of three miRNAs to up-regulate three targeted downstream mRNAs. The multiple circZNF800-miRNA-mRNA axes form chains of regulatory events likely to be related to enhanced expression of cancer stem cell markers and associated cellular functions. Our findings highlight the critical roles of circZNF800 in promoting cancer stem cell properties and contributing to the CRC tumorigenesis process. Silencing the circZNF800 suppresses the cancer stem cell phenotype in vitro and impedes tumor growth in mice and may form the basis for developing strategies for therapeutic treatment of colorectal cancer.

### Supplementary Information

**Additional file 1: Suppl. file 1: Table S1.** List of PCR primers and probes used.

** Additional file 2: Suppl. file 2: Table S2.** Expression profiling of circZNF800 isoforms in cancer and non-cancer cells and tissues.

** Additional file 3: Suppl. file 3: Fig. S1.** Uncropped and unedited RNA gel electrophoresis for in vitro transcribed and circularized circZNF800.

** Additional file 4: Suppl. file 4: Fig. S2.** CircZNF800-miRNA & miRNA-mRNA seed sequence alignments.

## Data Availability

All analyzed data are included in this published article and its supplementary information files. The original data are available upon reasonable request to the corresponding authors.

## Abbreviations

ACVR1C: Activin A Receptor Type 1C
AKT: AKT serine/threonine kinase
ALK7: Activin A receptor type 1C
circRNA: Circular RNA
circZNF800: Circular RNA ZNF800
CRC: Colorectal cancer
CRISPR: Clustered Regularly Interspaced Short Palindromic Repeats
crRNA: CRISPR RNA
crSC: CRISPR RNA with scrambled sequence
Cy-5: Cyanine-5
EGFP: Enhanced green fluorescence protein
FACS: Fluorescence Activated Cell Sorter
FISH: *in situ* Hybridization
FZD3: Frizzled class receptor 3
GPRC5A: G protein-coupled receptor class C group 5 member A
ISC: Intestinal stem cell
KD: Knockdown
LGR5: Leucine rich repeat containing G protein-coupled receptor 5
miRNA: MicroRNA
NOTCH1: Notch receptor 1
OE: Over-expression
SOX9: SRY-box transcription factor 9
UTR: Untranslated region
VAPA: VAMP associated protein A
WNT5A: WNT family member 5A
ZNF800: Zinc-finger protein 800
