# Supplementary material for: Circular RNA ZNF800 (hsa_circ_0082096) regulates cancer stem cell properties and tumor growth in colorectal cancer
Source: BMC Cancer. 2023 Nov 10;23:1088. doi: 10.1186/s12885-023-11571-1 (PMC10636831; doi:10.1186/s12885-023-11571-1)
Supplement: Supplementary file 1 — Additional file 1: Suppl. file 1: Table S1. List of PCR primers and probes used. [file 12885_2023_11571_MOESM1_ESM.docx]

**Supplementary Table S1. List of PCR primers and probes used**

1. **Primers of qRT-PCR analysis of mRNA expression levels**

| **Target** | **Forward primer**  **(5’ – 3’)** | **Reverse primer**  **(5’ – 3’)** |
| --- | --- | --- |
| CircZNF800 | GGCTCTTGTCTGGAACTAAAC | TCTGAAGAGACTGCGGCATA |
| ZNF800 | ACGGATGCTGTGAACCAGTTTAT | TGGATGTCTGTAGTGGTTGCTG |
| OCT4 | AACCTGGAGTTTGTGCCAGGGTTT | TGAACTTCACCTTCCCTCCAACCA |
| SOX2 | AGAAGAGGAGAGAGAAAGAAAGGGA | GAGAGAGGCAAACTGGAATCAGGATC |
| KLF4 | CATCTCAAGGCACACCTGCGAA | TCGGTCGCATTTTTGGCACTGG |
| CMYC | CCTGGTGCTCCATGAGGAGAC | CAGACTCTGACCTTTTGCCAGG |
| NANOG | TTTGTGGGCCTGAAGAAAACT | AGGGCTGTCCTGAATAAGCAG |
| GAPDH | CTCAACTACATGGTTTACATGTTC | TGGAAGATGGTGATGGGATT |

**B. Primers for miRNA stem-loop qRT-PCR**

| **MicroRNA** | **Stem-loop RT primer (5’ – 3’)** | **Forward primer (5’ – 3’)** |
| --- | --- | --- |
| miR-140-3p | GTCGTATCCAGTGCAGGGTCCGAGGTATTCGCACTGGATACGACGGCACCA | AATACGCGTACCACAGGGTAG |
| miR-579-3p | GTCGTATCCAGTGCAGGGTCCGAGGTATTCGCACTGGATACGACAATCGCG | ATGCGCGCTTCATTTGGTATAA |
| miR-382-5p | GTCGTATCCAGTGCAGGGTCCGAGGTATTCGCACTGGATACGACCGAATCC | AAGCCGCAGAAGTTGTTCGTG |

**C. Probes**

| **Probe** | **Sequence (5 – 3’)** |
| --- | --- |
| RNA FISH probe | (Cy5)CATCTTTTAATAAAATATGCTTAAGTTGTTTAGTTCCAGACAAGAGCCTTAGATCTTGT |
| CircZNF800 crRNA1 | AGTTGTTTAGTTCCAGACAAGAGCC |
| CircZNF800 crRNA2 | AGTTCCAGACAAGAGCCTTAGATCT |
