# Supplementary material for: Circular RNA ZNF800 (hsa_circ_0082096) regulates cancer stem cell properties and tumor growth in colorectal cancer
Source: BMC Cancer. 2023 Nov 10;23:1088. doi: 10.1186/s12885-023-11571-1 (PMC10636831; doi:10.1186/s12885-023-11571-1)
Supplement: Supplementary file 2 — Additional file 2: Suppl. file 2: Table S2. Expression profiling of circZNF800 isoforms in cancer and non-cancer cells and tissues. [file 12885_2023_11571_MOESM2_ESM.docx]

**Supplementary Table S2.** Expression profiling of circZNF800 isoforms in cancer and non-cancer cells and tissues^1^

| **circRNA ID** | **Spliced length (nt)** | **Exon/intron retained^2^** | **Cell types detected^3^** | | | **Reference** |
| --- | --- | --- | --- | --- | --- | --- |
|  |  |  | **Cancer** | **Non-cancer** | **Brain tissues** |  |
| hsa_circ_0082095 | 2,352 | 4, 5, intr. 5 (p) | K562, SK-N-SH-RA | NHEK | NA | [24] |
| hsa_circ_0082096  (circZNF800) | 1,837 | 4 & 5 | K562, CRC-derived spheroids | AG04450, LCL, chondrocytes | Multiple brain tissues | [19, 24-26] |
| hsa_circ_0082097 | 1,933 | 3 - 5 | SK-N-SH-RA | NA | Cerebellum, occipital lobe | [24, 25] |
| hsa_circ_0082098 | 2,052 | 2 - 5 | NA | BJ | NA | [24] |
| hsa_circ_0082099 | 359 | 2 - 4 | SK-N-SH-RA | NA | NA | [24] |
| hsa_circ_0133240 | 249 | Intr. 6 (p) | NA | NA | Occipital lobe | [25] |
| hsa_circ_0133241 | 1,927 | 4, 5, intr. 6 (p) | NA | NA | Sy5y | [25] |
| hsa_circ_0133242 | 630 | 3, intr. 3 (p) | NA | NA | Frontal cortex | [25] |

^1^Adapted from circBase (<http://www.circbase.org>; Feb 28, 2023). ^2^In all cases, only partial (p) intron (intr.) sequences are retained. ^3^K562, chronic myeloid leukemia cells; SK-N-SH-RA, neuroblastoma SK-N-SH cells differentiated with retinoic acid; CRC, colorectal cancer; NHEK, normal human epidermal keratinocyte; AG04450, fetal lung fibroblast; BJ, LCL, EBV-transformed lymphoblastoid cells; Sy5y, thrice-subcloned cell line derived from the SK-N-SH neuroblastoma cell line. NA, not available.
