# Supplementary material for: Circular RNA ZNF800 (hsa_circ_0082096) regulates cancer stem cell properties and tumor growth in colorectal cancer
Source: BMC Cancer. 2023 Nov 10;23:1088. doi: 10.1186/s12885-023-11571-1 (PMC10636831; doi:10.1186/s12885-023-11571-1)
Supplement: Supplementary file 3 — Additional file 3: Suppl. file 3: Fig. S1. Uncropped and unedited RNA gel electrophoresis for in vitro transcribed and circularized circZNF800. [file 12885_2023_11571_MOESM3_ESM.pptx]

## Slide 1
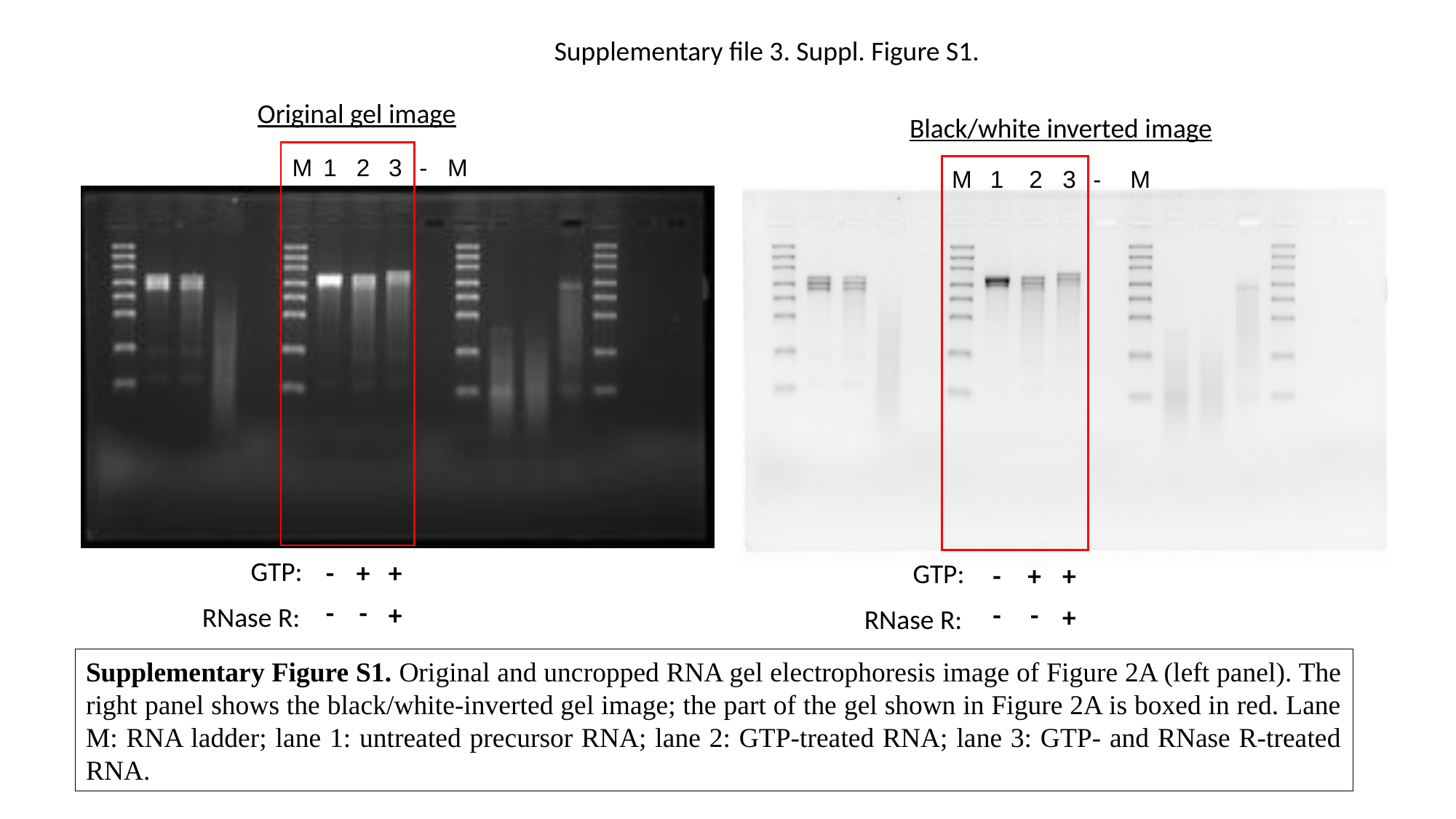

Supplementary file 3. Suppl. Figure S1.
Original gel image
Black/white inverted image
M
1
2
3
-
M
GTP:
-
+
+
-
-
+
RNase R:
M
1
2
3
-
M
GTP:
-
+
+
-
-
+
RNase R:
Supplementary Figure S1. Original and uncropped RNA gel electrophoresis image of Figure 2A (left panel). The right panel shows the black/white-inverted gel image; the part of the gel shown in Figure 2A is boxed in red. Lane M: RNA ladder; lane 1: untreated precursor RNA; lane 2: GTP-treated RNA; lane 3: GTP- and RNase R-treated RNA.
