# Supplementary material for: Circular RNA ZNF800 (hsa_circ_0082096) regulates cancer stem cell properties and tumor growth in colorectal cancer
Source: BMC Cancer. 2023 Nov 10;23:1088. doi: 10.1186/s12885-023-11571-1 (PMC10636831; doi:10.1186/s12885-023-11571-1)
Supplement: Supplementary file 4 — Additional file 4: Suppl. file 4: Fig. S2. CircZNF800-miRNA & miRNA-mRNA seed sequence alignments. [file 12885_2023_11571_MOESM4_ESM.pptx]

## Slide 1
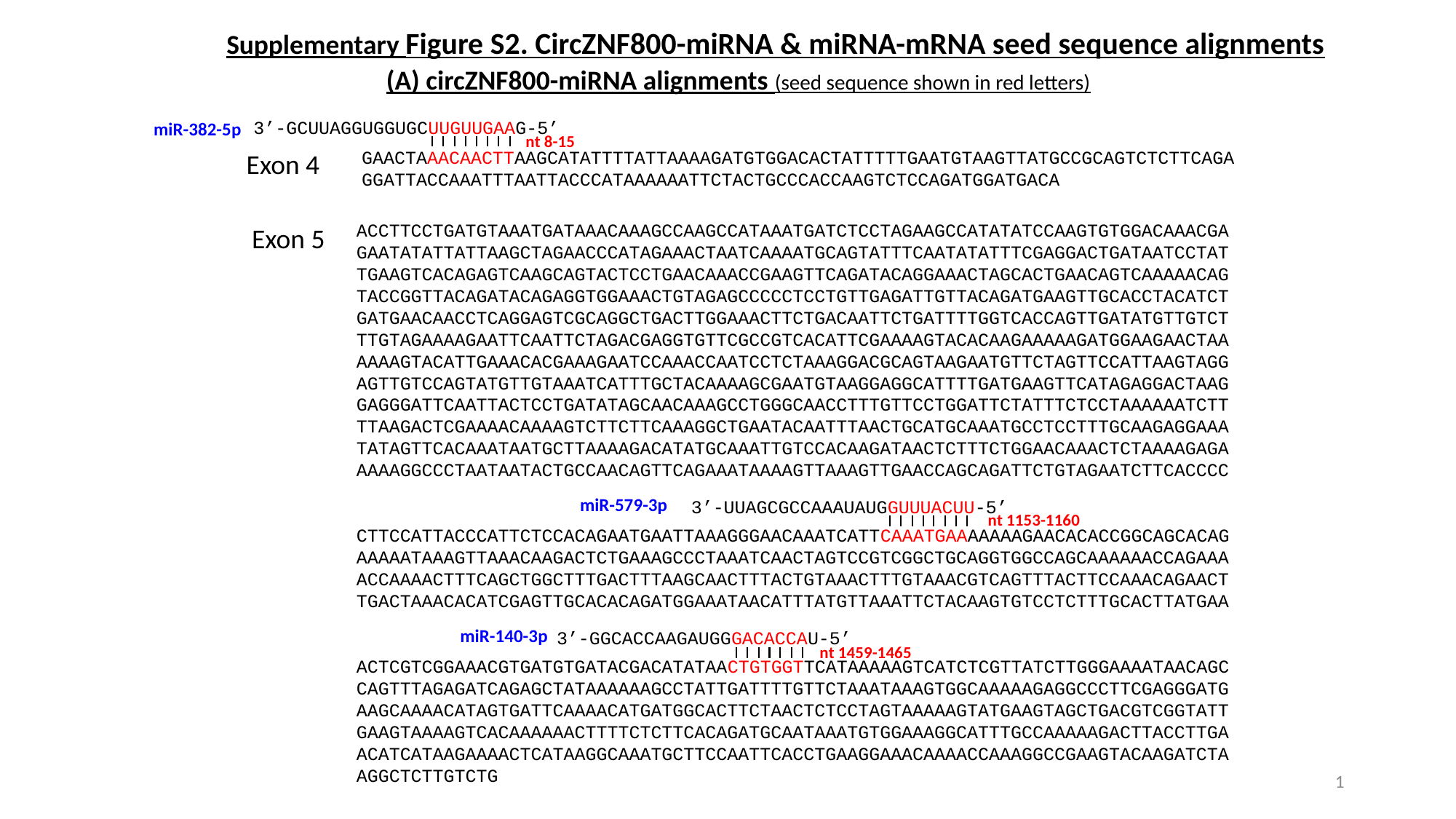

Supplementary Figure S2. CircZNF800-miRNA & miRNA-mRNA seed sequence alignments
(A) circZNF800-miRNA alignments (seed sequence shown in red letters)
3’-GCUUAGGUGGUGCUUGUUGAAG-5’
miR-382-5p
nt 8-15
GAACTAAACAACTTAAGCATATTTTATTAAAAGATGTGGACACTATTTTTGAATGTAAGTTATGCCGCAGTCTCTTCAGA
GGATTACCAAATTTAATTACCCATAAAAAATTCTACTGCCCACCAAGTCTCCAGATGGATGACA
Exon 4
ACCTTCCTGATGTAAATGATAAACAAAGCCAAGCCATAAATGATCTCCTAGAAGCCATATATCCAAGTGTGGACAAACGA
GAATATATTATTAAGCTAGAACCCATAGAAACTAATCAAAATGCAGTATTTCAATATATTTCGAGGACTGATAATCCTAT
TGAAGTCACAGAGTCAAGCAGTACTCCTGAACAAACCGAAGTTCAGATACAGGAAACTAGCACTGAACAGTCAAAAACAG
TACCGGTTACAGATACAGAGGTGGAAACTGTAGAGCCCCCTCCTGTTGAGATTGTTACAGATGAAGTTGCACCTACATCT
GATGAACAACCTCAGGAGTCGCAGGCTGACTTGGAAACTTCTGACAATTCTGATTTTGGTCACCAGTTGATATGTTGTCT
TTGTAGAAAAGAATTCAATTCTAGACGAGGTGTTCGCCGTCACATTCGAAAAGTACACAAGAAAAAGATGGAAGAACTAA
AAAAGTACATTGAAACACGAAAGAATCCAAACCAATCCTCTAAAGGACGCAGTAAGAATGTTCTAGTTCCATTAAGTAGG
AGTTGTCCAGTATGTTGTAAATCATTTGCTACAAAAGCGAATGTAAGGAGGCATTTTGATGAAGTTCATAGAGGACTAAG
GAGGGATTCAATTACTCCTGATATAGCAACAAAGCCTGGGCAACCTTTGTTCCTGGATTCTATTTCTCCTAAAAAATCTT
TTAAGACTCGAAAACAAAAGTCTTCTTCAAAGGCTGAATACAATTTAACTGCATGCAAATGCCTCCTTTGCAAGAGGAAA
TATAGTTCACAAATAATGCTTAAAAGACATATGCAAATTGTCCACAAGATAACTCTTTCTGGAACAAACTCTAAAAGAGA
AAAAGGCCCTAATAATACTGCCAACAGTTCAGAAATAAAAGTTAAAGTTGAACCAGCAGATTCTGTAGAATCTTCACCCC
CTTCCATTACCCATTCTCCACAGAATGAATTAAAGGGAACAAATCATTCAAATGAAAAAAAGAACACACCGGCAGCACAG
AAAAATAAAGTTAAACAAGACTCTGAAAGCCCTAAATCAACTAGTCCGTCGGCTGCAGGTGGCCAGCAAAAAACCAGAAA
ACCAAAACTTTCAGCTGGCTTTGACTTTAAGCAACTTTACTGTAAACTTTGTAAACGTCAGTTTACTTCCAAACAGAACT
TGACTAAACACATCGAGTTGCACACAGATGGAAATAACATTTATGTTAAATTCTACAAGTGTCCTCTTTGCACTTATGAA
ACTCGTCGGAAACGTGATGTGATACGACATATAACTGTGGTTCATAAAAAGTCATCTCGTTATCTTGGGAAAATAACAGC
CAGTTTAGAGATCAGAGCTATAAAAAAGCCTATTGATTTTGTTCTAAATAAAGTGGCAAAAAGAGGCCCTTCGAGGGATG
AAGCAAAACATAGTGATTCAAAACATGATGGCACTTCTAACTCTCCTAGTAAAAAGTATGAAGTAGCTGACGTCGGTATT
GAAGTAAAAGTCACAAAAAACTTTTCTCTTCACAGATGCAATAAATGTGGAAAGGCATTTGCCAAAAAGACTTACCTTGA
ACATCATAAGAAAACTCATAAGGCAAATGCTTCCAATTCACCTGAAGGAAACAAAACCAAAGGCCGAAGTACAAGATCTA
AGGCTCTTGTCTG
Exon 5
miR-579-3p
3’-UUAGCGCCAAAUAUGGUUUACUU-5’
nt 1153-1160
miR-140-3p
3’-GGCACCAAGAUGGGACACCAU-5’
nt 1459-1465
1

## Slide 2
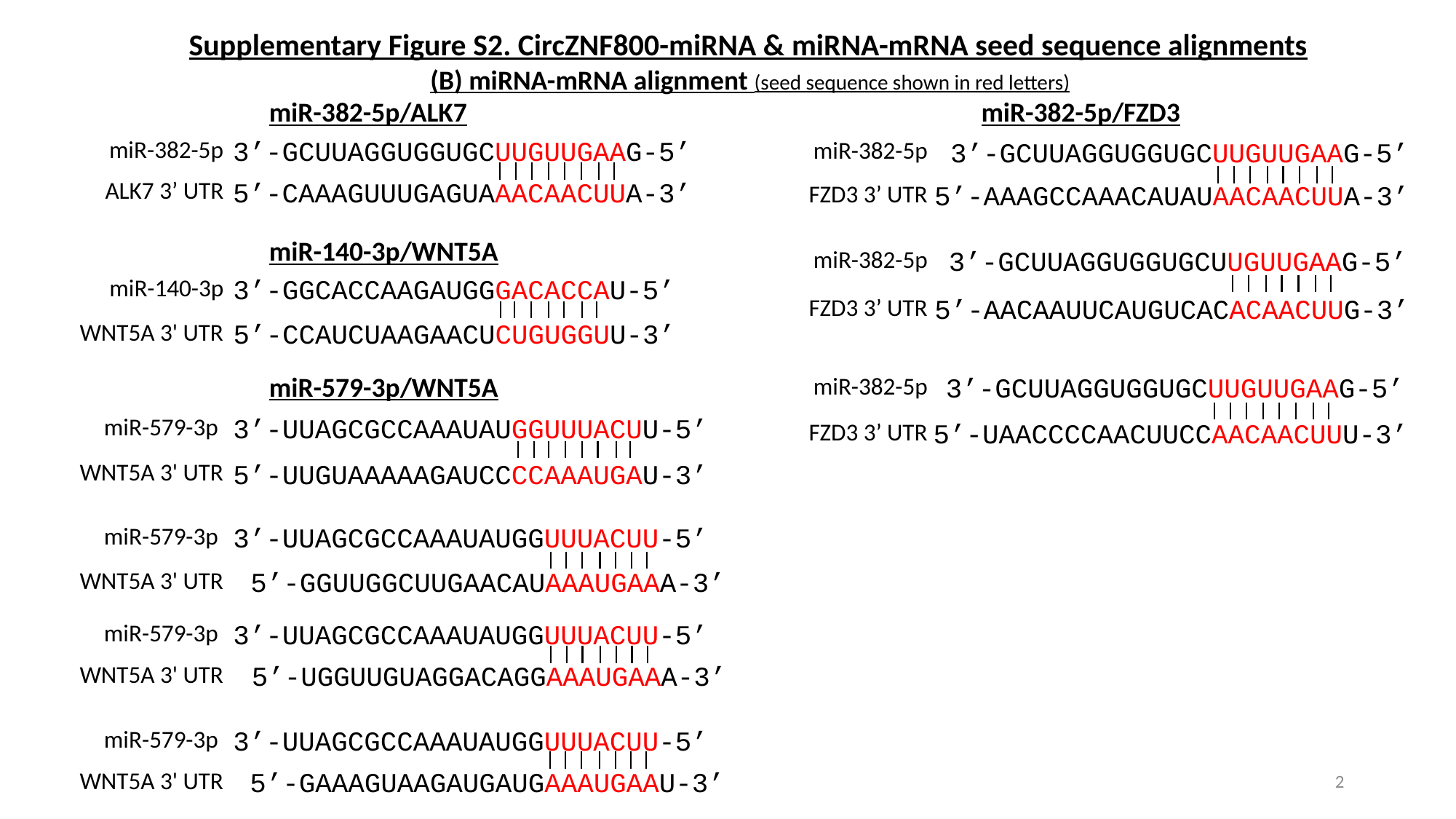

Supplementary Figure S2. CircZNF800-miRNA & miRNA-mRNA seed sequence alignments
(B) miRNA-mRNA alignment (seed sequence shown in red letters)
miR-382-5p/ALK7
miR-382-5p/FZD3
3’-GCUUAGGUGGUGCUUGUUGAAG-5’
 miR-382-5p
miR-382-5p
3’-GCUUAGGUGGUGCUUGUUGAAG-5’
ALK7 3’ UTR
5’-CAAAGUUUGAGUAAACAACUUA-3’
FZD3 3’ UTR
5’-AAAGCCAAACAUAUAACAACUUA-3’
miR-140-3p/WNT5A
3’-GCUUAGGUGGUGCUUGUUGAAG-5’
miR-382-5p
miR-140-3p
3’-GGCACCAAGAUGGGACACCAU-5’
5’-AACAAUUCAUGUCACACAACUUG-3’
FZD3 3’ UTR
5’-CCAUCUAAGAACUCUGUGGUU-3’
WNT5A 3' UTR
miR-579-3p/WNT5A
miR-382-5p
3’-GCUUAGGUGGUGCUUGUUGAAG-5’
3’-UUAGCGCCAAAUAUGGUUUACUU-5’
miR-579-3p
FZD3 3’ UTR
5’-UAACCCCAACUUCCAACAACUUU-3’
WNT5A 3' UTR
5’-UUGUAAAAAGAUCCCCAAAUGAU-3’
3’-UUAGCGCCAAAUAUGGUUUACUU-5’
miR-579-3p
WNT5A 3' UTR
5’-GGUUGGCUUGAACAUAAAUGAAA-3’
miR-579-3p
3’-UUAGCGCCAAAUAUGGUUUACUU-5’
5’-UGGUUGUAGGACAGGAAAUGAAA-3’
WNT5A 3' UTR
3’-UUAGCGCCAAAUAUGGUUUACUU-5’
miR-579-3p
2
5’-GAAAGUAAGAUGAUGAAAUGAAU-3’
WNT5A 3' UTR

## Slide 3
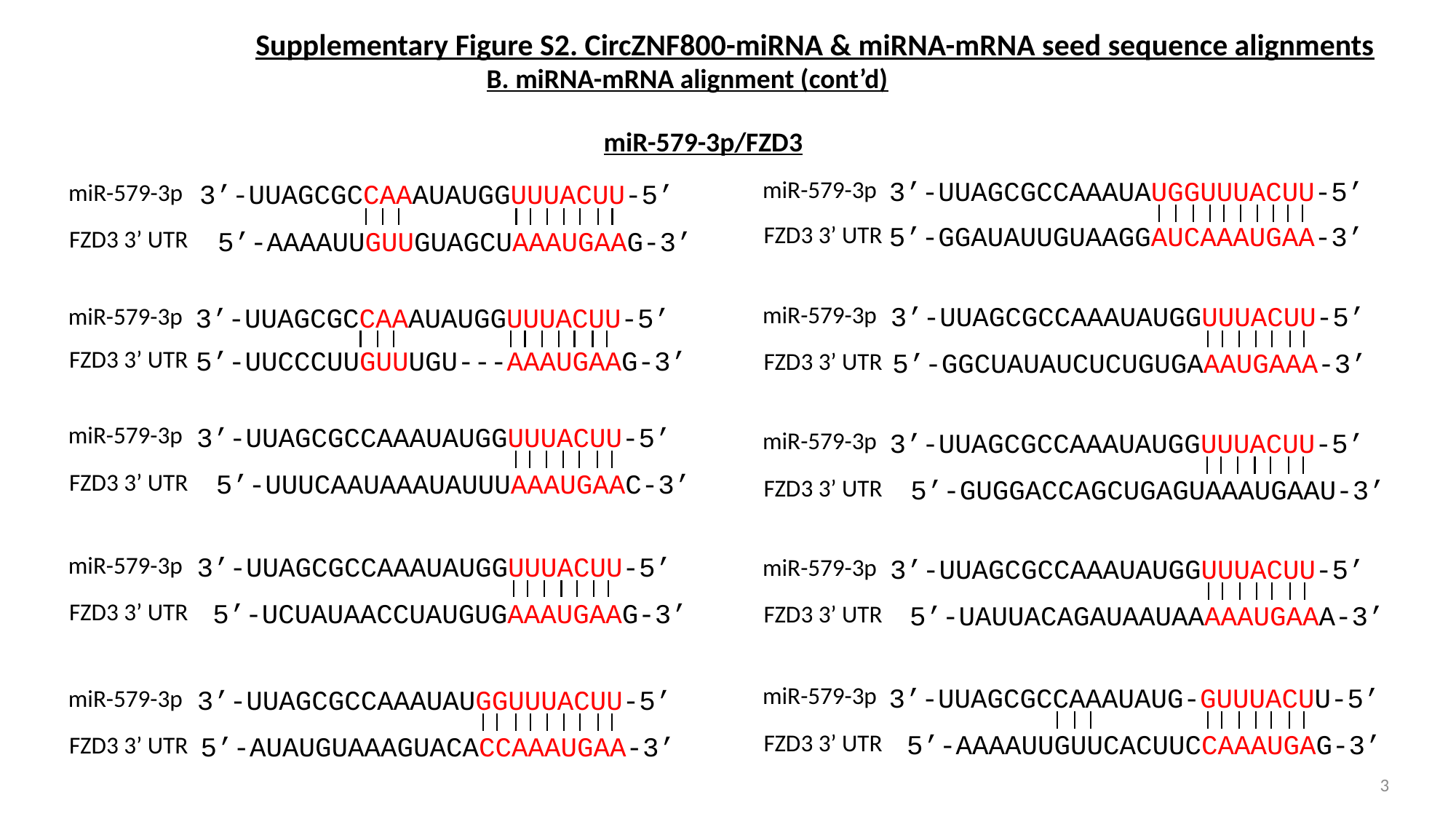

Supplementary Figure S2. CircZNF800-miRNA & miRNA-mRNA seed sequence alignments
B. miRNA-mRNA alignment (cont’d)
miR-579-3p/FZD3
3’-UUAGCGCCAAAUAUGGUUUACUU-5’
miR-579-3p
3’-UUAGCGCCAAAUAUGGUUUACUU-5’
miR-579-3p
FZD3 3’ UTR
5’-GGAUAUUGUAAGGAUCAAAUGAA-3’
FZD3 3’ UTR
5’-AAAAUUGUUGUAGCUAAAUGAAG-3’
miR-579-3p
3’-UUAGCGCCAAAUAUGGUUUACUU-5’
3’-UUAGCGCCAAAUAUGGUUUACUU-5’
miR-579-3p
5’-UUCCCUUGUUUGU---AAAUGAAG-3’
FZD3 3’ UTR
5’-GGCUAUAUCUCUGUGAAAUGAAA-3’
FZD3 3’ UTR
3’-UUAGCGCCAAAUAUGGUUUACUU-5’
miR-579-3p
miR-579-3p
3’-UUAGCGCCAAAUAUGGUUUACUU-5’
5’-UUUCAAUAAAUAUUUAAAUGAAC-3’
FZD3 3’ UTR
5’-GUGGACCAGCUGAGUAAAUGAAU-3’
FZD3 3’ UTR
miR-579-3p
3’-UUAGCGCCAAAUAUGGUUUACUU-5’
3’-UUAGCGCCAAAUAUGGUUUACUU-5’
miR-579-3p
5’-UCUAUAACCUAUGUGAAAUGAAG-3’
FZD3 3’ UTR
FZD3 3’ UTR
5’-UAUUACAGAUAAUAAAAAUGAAA-3’
miR-579-3p
3’-UUAGCGCCAAAUAUG-GUUUACUU-5’
3’-UUAGCGCCAAAUAUGGUUUACUU-5’
miR-579-3p
5’-AAAAUUGUUCACUUCCAAAUGAG-3’
FZD3 3’ UTR
5’-AUAUGUAAAGUACACCAAAUGAA-3’
FZD3 3’ UTR
3
